# Effectiveness of Physical Exercise to Reduce Cardiovascular Risk Factors in Youths: A Randomized Clinical Trial

**DOI:** 10.14740/jocmr1700w

**Published:** 2015-03-01

**Authors:** Claudia Ciceri Cesa, Sandra Mari Barbiero, Rosemary de Oliveira Petkowicz, Carla Correa Martins, Renata das Virgens Marques, Allana Abreu Martins Andreolla, Lucia Campos Pellanda

**Affiliations:** a Instituto de Cardiologia, Fundacao Universitaria de Cardiologia, Avenida Princesa Isabel, 370, 3º andar, Porto Alegre, Rio Grande do Sul 90620-000, Brazil; b Universidade Federal de Ciencias da Saude de Porto Alegre, Avenida Osvaldo Aranha, 245, Porto Alegre, Rio Grande do Sul 90050-170, Brazil

**Keywords:** Exercise, Children, Risk factors, Atherosclerosis

## Abstract

**Background:**

The aim of the current study was to test the effectiveness of a physical activity and exercise-based program in a clinical context to reduce cardiovascular risk factors in children and adolescents.

**Methods:**

A randomized clinical trial was conducted in a pediatric preventive outpatient clinic. Intervention was 14 weeks of exercise for the intervention group or general health advice for the control group. The primary and the secondary outcomes were reduction of cardiovascular risk factors and the feasibility and the effectiveness of clinical advice plan to practice physical exercises at home.

**Results:**

A total of 134 children were screened; 26 met eligibility criteria. Of these, 10 were allocated in the exercise intervention group and nine were included in the control group until the end of the intervention. Those patients who discontinued the intervention had the lowest scores of z-BMI (P = 0.033) and subscapular skin fold (P = 0.048). After 14 weeks of intervention, no statistical differences were found between the groups. High-density lipoprotein cholesterol (HDL-C) was higher in the exercise group, with a mild tendency to be significant (P = 0.066). Patients who adhere to treatment had diastolic blood pressure decreased from baseline to the end of the follow-up period in the control group (P = 0.013). Regardless of this result, the other comparisons within the group were not statistically different between T0 and T14.

**Conclusion:**

A low-cost physical activity advice intervention presented many barriers for implementation in routine clinical care, limiting its feasibility and evaluation of effectiveness to reduce cardiovascular risk factors.

## Introduction

The prevalence of cardiovascular risk factors in childhood is increasing all over the world. Overweight and obesity prevalence rates among children increased from 26.9 million (4.2%) in 1990 to 42.8 million (6.7%) in 2010 worldwide [1]. Trends in blood pressure (BP) showed increase of 0.8 mm Hg from 2005 to 2010 in Chinese children [2]. Systolic levels are 13 mm Hg higher in UK children over time [3], and less than 70% of US youths exhibited ideal total cholesterol [4].

It has been shown that a combination of risk factors including high levels of body mass index (BMI), lipids, and BP at 9 years of age predicts atherosclerosis in adulthood [5].

Primordial and primary preventive interventions to reduce cardiovascular risk factors in youths are an important target in the health system to try to avoid cardiovascular outcomes in the future. Several randomized clinical trials (RCTs) were designed to reduce body weight in childhood. However, RCTs under real-life conditions, with intervention target to reduce childhood cardiovascular risk factors in routine pediatric outpatient care are rare [6, 7].

Physical activity target for youths is 60 min/day of moderate-to-vigorous physical activity [8]. Even with guidance to be physically active, no guideline provides information on how to manage or implement physical activity and exercise advice in the clinical practice. The intensity of the interventions and the health professionals involved in the clinical approach vary widely from one treatment center to another [6].

Systematic reviews have revealed that a combination of physical activity and diet interventions provided significant reductions in BMI [9-11]. Nevertheless, these results provided information about the general school-age populations and not an intervention targeted to patients in clinical care. An observational study, based on a 2-year follow-up of pediatric obesity care centers using lifestyle intervention approach, found that 22% of youths reduced their body mass status after 6 months, 15% after 12 months, and only 7% after 24 months when performing intention-to-treat analysis [6]. Conversely, when the authors analyzed only the children who completed 24 months of intervention, they found reductions of 83%, 82%, and 76%, respectively. Thus, compliance with the recommendations is paramount to achieve the desired effect, but it is the most challenging aspect of physical activity or other lifestyle modification intervention. Lifestyle interventions including exercise and diet are effective to improve cardio-metabolic risk factors in obese children [10]; however, the feasibility of physical exercise advice in clinical care remains unclear.

The aim of the present study was to test the effectiveness of a physical activity and exercise-based program in the routine practice of a clinical context to improve cardiovascular risk factors: BMI, BP, lipids and lipoproteins (total cholesterol (TC), low-density lipoprotein cholesterol (LDL-C), high-density lipoprotein cholesterol (HDL-C), triglycerides (TG), glucose, and high-sensitivity C-reactive protein (hs-CRP)) in children and adolescents.

## Methods

### Participants

Children and adolescents were recruited to participate by means of advertisements on the television, newspapers, and the radio from September 2009 to April 2011. Inclusion criteria were children and adolescents aged 6 - 17 years, with at least two of the following health conditions: overweigh or obesity, high BP, high TC, or sedentary lifestyle (no regular physical activity). Exclusion criteria were medical conditions that would limit physical exercise and regular use of medications (for any medical condition). Written informed consent was obtained from parents and informed assent from children. The study was conducted at the Instituto de Cardiologia do Rio Grande do Sul, Fundacao Universitaria de Cardiologia, Porto Alegre, Brazil (population: 1,409,351, area: 496.862 km^2^) [12]. The Institutional Review Board approved the study protocol under the number 2994-01. ClinicalTrial.gov Identifier: NCT01580319.

### Procedure

A single-blinded (outcome assessor), prospective, parallel, randomized controlled clinical trial was conducted in a pediatric preventive outpatient clinic of a tertiary referral hospital for heart diseases. A sample size of 90 patients (45 each group) was calculated based on reductions observed in TC in patients from PREVINA (pediatric preventive cardiology outpatient clinic in the same hospital) from 215.4 ± 41.83 mg/dL to 190.60 ± 34.57 mg/dL after 3 months of physical activity advice (pilot trial, no randomization at this level). We ran a comparison of means for two independent samples, with α of 0.05 and a desired power of 0.80, using the online tool made available by the University of British Columbia, Department of Statistics at http://www.stat.ubc.ca/~rollin/stats/ssize/n2.html.

Patients were randomly assigned to the exercise intervention group or to the control group. A randomization list was generated by an external researcher on the website Randomization.com [13], a web-based random number generator. The list included nine random blocks of 10 patients each to ensure equal distribution across the arms. Allocations were concealed in opaque and sealed envelopes until baseline tests were completed. The first visit included explanation about the research protocol, written informed consent, physical examination, collection of anthropometric data, and blood test order. Second visit consisted of delivering blood test results and providing participants with information about allocation to exercise intervention or control group. All children complied with the visit schedule (exercise group and control group) once a month. The first visit was related to enrolment-related aspects. The second visit was time “zero week” (T0) with allocation to exercise intervention group (first exercise plan to do at home) or control group (advice to reduce screen time to 2 h/day and continue their usual activities). The third visit was time “6 weeks” (T6) with the second exercise plan for the exercise intervention group or reinforcement about screen time and usual activities for the control group. The fourth visit was time “10 weeks” (T10) with the third exercise plan for the exercise intervention group or reinforcement about screen time and usual activities for the control group. The fifth visit was time “14 weeks” (T14) with anthropometric measurements and blood test. After 3 months of intervention, children were followed up to check physical activity levels at the sixth visit or “27 weeks” (6 months after T0). Between each visit to the clinic, we called the participants once a week to check if they were implementing the intervention plan (exercise group) or if they changed their lifestyle (control group). If the participants discontinued the treatment for any reason, the team of researchers tried to help the family to find a solution for them to adhere to the intervention plan.

The patients and the researchers were aware of the intervention allocation because exercise interventions are hard to blind or develop placebo (even low intensity or low volume of exercises can cause health related improvements). In order to improve the methodological strength of the study, randomization allocations were concealed until baseline tests were completed and the outcome assessor was blind to the intervention assignment.

### Exercise intervention group

A general plan of physical activities and exercises was developed for each month of intervention (three plans) with physical activities, exercises and games to pay at home, three times a week per 50 min each section (150 min/week).

The Borg’s scale of perceived exertion CR-10 (10 point scale: 0 - nothing at all; 0.5 - extremely weak; 1 - very weak; 2 - weak; 3 - moderate; 5 - strong; 7 - very strong; 10 - extremely strong; maximal) [14] was used to assess correct exercise intensity. Physical activities were planned to develop gross and fine motor skills, with enjoyment and fun basis to promote cardiovascular fitness and skills enhancement. Each exercise plan was divided in 7 min to warm-up (Borg’s scale from 2 to 3), 15 min to motor-skilled exercises (Borg’s scale at 3), 20 min to fitness (Borg’s scale from 5 to 9), 5 min to calm down (Borg’s scale from 1 to 2), and 3 min to organize material. Families were encouraged to promote a physically active lifestyle (e.g., go walking to school) and go to parks to play with the children on weekends.

### Measurements and classifications

In the first visit (T0), children and parents were asked about demographic data (age and gender), had their anthropometric data measured (parents: weight and height; children: weight, height, abdominal circumference, waist circumference (WC) (tricipital, subscapular, and abdominal), skin folds), and underwent physical examination (general health condition, and heart auscultation), BP (systolic blood pressure (SBP), and diastolic blood pressure (DBP)), blood tests (complete blood work, TC, LDL-C, HDL-C, TG, fasting glucose test, and hs-CRP), and screen time (television, videogame, and personal computer).

Weight and height were measured according to the World Health Organization’s (WHO) guidance. Weight was measured using a digital scale (precision: 0.1 kg), and height was measured using a stadiometer (precision: 0.5 cm) (barefoot and light clothes). Overweight and obesity were considered if BMI z-score (z-BMI) is > +1 standard deviation (SD) and > +2 SD, respectively, by Anthroplus software (provided by WHO and based on the WHO Reference 2007 growth charts) [15-18]. WC was measured using a tape measure at the uppermost lateral border of the hip crest (ilium). WC was classified according to the National Health and Nutrition Examination Survey 2003 - 2006 (NAHES 2003 - 2006) tables as < 90th or ≥ 90th percentile by gender and age [19].

The measurements of BP were according to The Fourth Report on the Diagnosis, Evaluation and Treatment of High Blood Pressure in Children and Adolescents in accordance with pediatric guidelines [8, 20, 21]. BP levels labels were normotensive (SBP and DBP < 90th percentile), prehypertension (pre-HTN) (SBP and/or DBP ≥ 90th percentile < 95th percentile or ≥ 120/80 mm Hg), stage 1 hypertension (stage 1 HTN) (SBP and/or DBP ≥ 95th percentile < 99th percentile + 5 mm Hg), or stage 2 hypertension (stage 2 HTN) (SBP and/or DPB percentiles ≥ 99th percentile + 5 mm Hg) [8, 20, 21].

Blood test results were classified according to pediatric guidelines [8, 21]. Lipid panel included: TC (acceptable (< 170 mg/dL), borderline-high (170 - 199 mg/dL), high (≥ 200 mg/dL)); LDL-C (acceptable (< 110 mg/dL), borderline-high (110 - 129 mg/dL), high (≥ 130 mg/dL)); HDL-C (low (< 40 mg/dL), acceptable (≥ 45 mg/dL), borderline-high (40 - 45 mg/dL)); and TG (0 - 9 years: acceptable (< 75 mg/dL), borderline-high (75 - 99 mg/dL), high (≥ 100 mg/dL), 10 - 19 years: acceptable (< 90 mg/dL), borderline-high (90 - 129 mg/dL), high (≥ 130 mg/dL)). Fasten glucose was defined as acceptable (< 100 mg/dL) and high (≥ 100 mg/dL).

Screen time was categorized in low levels of screen time (< 120 min/day) or high levels (≥ 120 min/day) according to the Expert Panel on Integrated Guidelines for Cardiovascular Health and Risk Reduction in Children and Adolescents: Summary Report of the AAP [8] and the I Guidelines of Prevention of Atherosclerosis in Childhood and Adolescence [21]. The international physical activity questionnaire (IPAQ) was used at the first visit (T0) to assess the physical activity level [22].

For the intervention group (exercise group), compliance was determined if patients followed the intervention protocol at least three times a week and attended the monthly visits. For the control group, adherence was determined if participants came to the monthly visit.

### Outcomes

The primary outcome was the intention-to-treat analysis of cardiovascular risk factor reductions based on improvements of BMI, BP, lipids and lipoproteins (TC, LDL-c, HDL-c, and TG), glucose, and hs-CRP.

The secondary outcome was the feasibility and the effectiveness (for adherent patients) of a clinical advice plan using low cost and recycled materials to do exercise at home.

### Statistical methods

We performed a descriptive analysis to describe the population with central tendency and dispersion for continuous variables and proportions for categorical variables (total number and prevalence). To compare the means between the exercise intervention group and the control group at T0 and T14, we used independent-samples *t*-test. Mean differences within the groups from T0 to T14 were calculated using paired-samples *t*-test. Nonparametric tests were used to confirm *t*-test results in asymmetric distributions (nonparametric test independent samples for differences between the groups (T0 and T14) and related samples for differences within the groups (from T0 to T14)). Pearson’s Chi-square test or Fisher’s exact test was used to determine associations for categorical variables. Linear-by-linear association was used for ordinal variables. Fisher’s exact test was used to determine associations if cell contained less than five counts. General linear model analyses were run to verify if endpoint variables were statistically significant after baseline adjustments. Statistical analyses were performed using SPSS for Macintosh, version 20.0. Statistical associations were considered P < 0.05.

## Results

**Figure 1:**
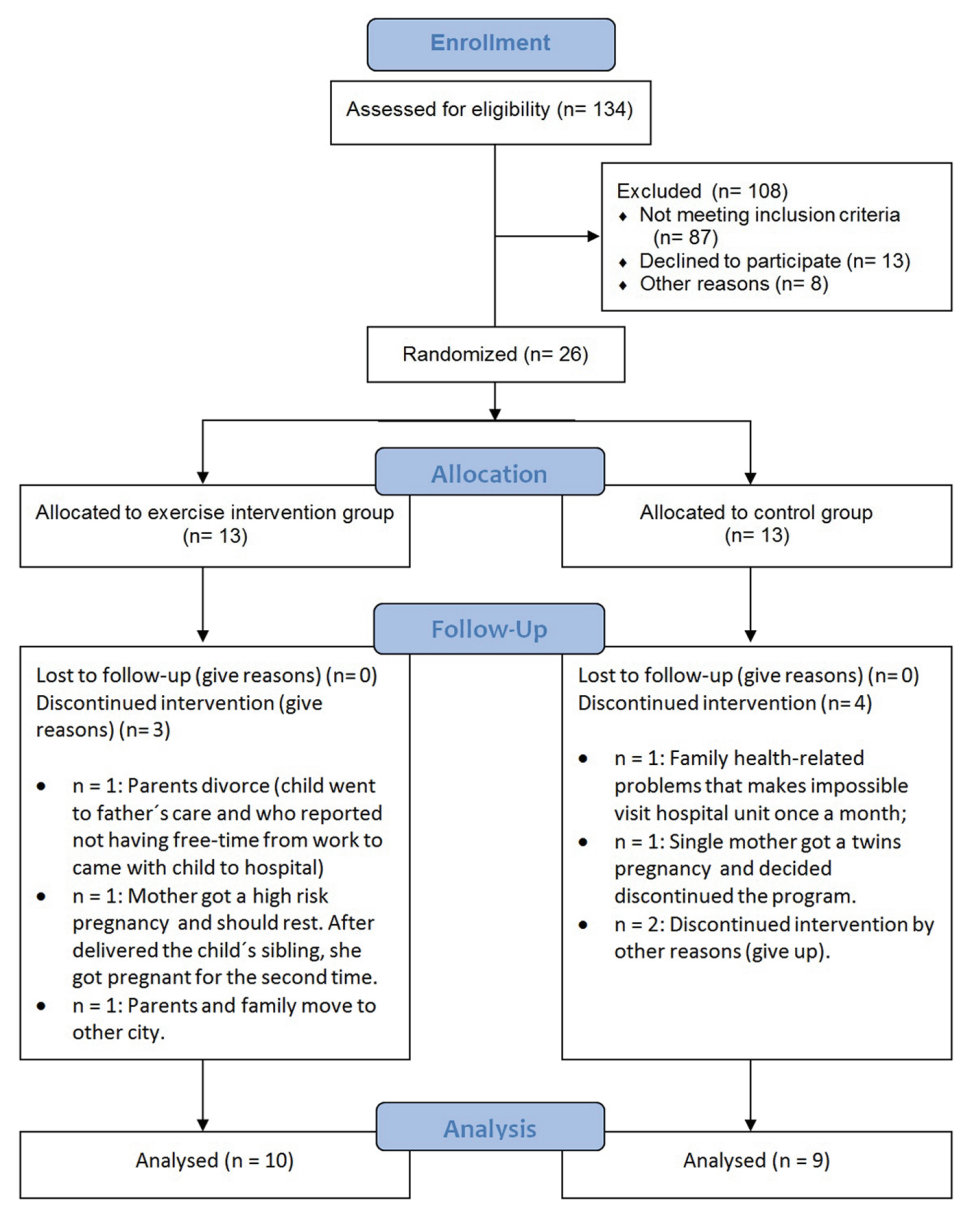
Participants flow diagram.

Figure 1 provides information about the participants’ flow. Although the calculated sample size was 90 patients, we were not able to enroll more than 26 patients. We advertised the study on television, newspapers, and the radio from September 2009 to April 2011 every fortnight (except for holidays). In average, every time we advertised the study, 3.5 families (parent and children) showed up. A total of 134 children were screened and 26 met the eligibility criteria. Of those, 10 of the participants allocated to the exercise intervention group and nine included in the control group remained until the end of the study. We analyzed differences and predictors for those who dropped out or remained in the trial. The patients who discontinued the treatment had the lowest scores of z-BMI (z-BMI lost to follow-up: 2.03 ± 0.99 vs. z-BMI kept in the trial: 3.05 ± 1.03; P = 0.033) and subscapular skin fold (subscapular skin fold lost to follow-up: 20.4 ± 14.0 mm vs. subscapular skin fold kept in the trial: 31.8 ± 11.5 mm; P = 0.048). Figure 1 provides all causes of dropout, but the main reasons for discontinuation were family issues (divorce, pregnancy, and family health-related problems).

The baseline characteristics were similar between the exercise intervention group and the control group (Table 1). After 14 weeks of intervention, no statistical differences were found between the exercise and the control group (Table 2). HDL-C was higher in the exercise group than in the control group, with a mild tendency to be significant (exercise group: 46.6 ± 8.9 mg/dL vs. control group: 39.6 ± 4.9 mg/dL; P = 0.066). Mean comparisons including patients who adhere to treatment within the group revealed that DBP decreased from baseline to the end of the follow-up period in the control group (T0: 77.2 ± 10.3 mm Hg vs. T14: 67.0 ± 7.4 mm Hg; P = 0.013). Regardless of this result, the other comparisons were not statically different between T0 and T4.

**Table 1:** Baseline Group Characteristics (T0)

Characteristic	T0		P value^a^
	Exercise group (n = 13)	Control group (n = 13)	
Age at first visit (years)	12.3 ± 2.5	12.7 ± 3.1	0.735
Gender			1.00^b^
Male	3	4	
Female	10	9	
BMI father	29.1 ± 8.4	29.6 ± 6.7	0.873
BMI mother	28.3 ± 5.3	29.1 ± 3.8	0.903
Weight	74.0 ± 19.1	69.8 ± 20.4	0.597
Height	153.9 ± 11.4	152.6 ± 9.9	0.772
z-BMI (SD)	2.8 ± 0.8	2.6 ± 1.3	0.669
WC (cm)	98.1 ± 14.8	93.4 ± 14.3	0.288
Heart rate	80.6 ± 12.2	79.9 ± 11.7	0.895
Blood pressure			
SBP (mm Hg)	108.0 ± 11.6	117.0 ± 13.1	0.079
DBP (mm Hg)	67.3 ± 10.5	75.0 ± 11.5	0.089
Normal levels	9 (69.2)	8 (61.5)	1.00^b^
High levels	4 (30.8)	5 (38.5)	
TC (mg/dL)	155.7 ± 20.3	146.7 ± 21.2	0.280
LDL-C (mg/dL)	95.3 ± 19.1	87.6 ± 20.2	0.342
HDL-C (mg/dL)	42.6 ± 7.8	37.5 ± 8.4	0.126
TG (mg/dL)	89.5 ± 28.7	105.5 ± 52.2	0.379
Fasting glucose (mg/dL)	90.3 ± 5.7	89.0 ± 6.1	0.581
hs-CRP (mg/dL)	0.42 ± 0.40	0.29 ± 0.21	0.317
Screen time (min/day)	369.0 ± 177.7	513.7 ± 185.3	0.115
Sleep time (min/day)	557.0 ± 96.7	475.0 ± 128.5	0.132
IPAQ			
Active	7 (53.8)	8 (61.5)	1.00^b^
Sedentary	6 (46.1)	5 (38.4)	
Active leisure time (min/day)	444.0 ± 435.6	424.6 ± 396.6	0.921
Skin folds			
Abdominal (mm)	41.8 ± 15.5	38.1 ± 15.6	0.562
Triceps (mm)	33.0 ± 12.6	29.5 ± 12.3	0.483
Subscapular (mm)	31.1 ± 14.4	26.0 ± 11.5	0.359

BMI: body mass index; z-BMI: body mass index z-score; SD: standard deviation; WC: waist circumference; cm: centimeters; SBP: systolic blood pressure; DBP: diastolic blood pressure; mm Hg: millimeters of mercury; TC: total cholesterol; LDL-C: low-density lipoprotein; HDL-C: high-density lipoprotein; TG: triglycerides; mg/dL: milligram per deciliter; mmol/L: millimole per liter; hs-CRP: high-sensitivity C-reactive protein; min/day: minutes per day; IPAQ: international physical activity questionnaire; mm, millimeters. ^a^Determined using independent-samples *t*-test for continuous data. ^b^Determined using Pearson’s Chi-square test for categorized variables.

**Table 2:** Mean Differences (Mean ± Standard Deviation) Between Groups at the End of Intervention (T14)

Characteristic	T14		P value^a^
	Exercise group (n = 10)	Control group (n = 9)	
Weight	81.5 ± 13.7	76.1 ± 14.0	0.476
Height	158.5 ± 9.0	157.3 ± 6.5	0.775
z-BMI (SD)	2.8 ± 0.57	2.5 ± 0.60	0.352
WC (cm)	90.5 ± 8.3	89.1 ± 7.3	0.740
Heart rate	87.1 ± 14.0	79.7 ± 10.6	0.267
Blood pressure			
SBP (mm Hg)	114.6 ± 8.6	112.5 ± 13.7	0.745
DBP (mm Hg)	72.3 ± 8.9	67.0 ± 7.4	0.223
TC (mg/dL)	153.7 ± 20.8	157.3 ± 18.0	0.699
LDL-C (mg/dL)	87.4 ± 15.0	99.1 ± 22.5	0.244
HDL-C (mg/dL)	46.6 ± 8.9	39.6 ± 4.9	0.066
TG (mg/dL)	99.8 ± 47.7	91.0 ± 20.5	0.635
Fasting glucose (mg/dL)	88.5 ± 4.3	89.0 ± 7.2	0.858
hs-CRP (mg/dL)	0.42 ± 0.36	0.47 ± 0.28	0.741
Screen time (min/day)	347.5 ± 240.4	361.4 ± 151.0	0.901
Sleep time (min/day)	562.5 ± 89.8	547.1 ± 86.9	0.760
IPAQ*			
Active	5 (83.3)	4 (57.1)	0.559^b^
Sedentary	1 (16.7)	3 (42.9)	
Active leisure time (min/day)	424.1 ± 265.3	519.8 ± 540.1	0.702
Skin folds			
Abdominal	50.3 ± 14.3	48.8 ± 7.0	0.798
Triceps	37.6 ± 9.0	34.2 ± 7.1	0.426
Subscapular	39.0 ± 14.3	35.4 ± 10.5	0.588

SD: standard deviation; WC: waist circumference; cm: centimeters; SBP: systolic blood pressure; DBP: diastolic blood pressure; mm Hg: millimeters of mercury; TC: total cholesterol; LDL-C: low-density lipoprotein; HDL-C: high-density lipoprotein; TG: triglycerides; mg/dL: milligram per deciliter; hs-CRP: high-sensitivity C-reactive protein; min/day: minutes per day; IPAQ: international physical activity questionnaire; mm: millimeters. ^a^Determined by use of the independent-samples *t*-test to continuous data. ^b^Determined using Pearson’s Chi-square test for categorized variables.

The analyses using a general linear model with overall sample showed no statistical differences between or within T0 and T14. When we included only the patients who adhered to treatment in the analyses, the results remained non-significant.

## Discussion

The present study did not achieve sufficient power to reach a conclusion about the benefits of providing advice on physical exercise (to be performed at home, under real life conditions) in an outpatient clinic setting. However, our results provide information about the feasibility of this intervention and the factors affecting compliance. Based on the main results, our study showed that planning and implementing this kind of intervention in routine clinical care is a difficult task. The reasons for the difficulties enrolling participants deserve to be discussed and may provide useful insights for future studies.

Family involvement is very important to ensure adherence to treatment because family issues were mentioned by the participants as the main reason for non-participation. It is important to address this issue because family issues represent real life conditions and will likely influence any physical activity intervention planned in similar contexts.

Of the 13 patients allocated to the exercise group, 10 completed the trial, and only six of these complied with all recommendations. This fact provides useful insights about the feasibility of home-based programs without supervision. Studies in schools or with a more structured approach have shown better results. However, these studies were targeted at two different extremes of the obesity spectrum: healthy children in schools or obese children are highly structured settings. Our target population consisted of obese children in a clinical setting within the public health system with limited resources. Thus, we tried to design a very simple and low cost intervention to be performed at home.

Loss to follow-up was also a major concern in other studies with lifestyle interventions. Lison et al [7] performed an RCT with exercise intervention and Mediterranean diet for children and 1 h of educational session for parents. Patients who did not complete the treatment were a problem, and the authors addressed this issue by allocating an unequal number of participants to each group (45 and 41 to intervention groups and 24 to control group). Adolescents who failed to complete the intervention program were 18% in two randomized clinical trials of weight lost programs [23]. Loss to follow-up was also a major outcome in a large 2-year follow-up study including 129 pediatric obesity care centers care [6].

High rates of dropout seem more challenging in children than in adults. Reasonable explanations are “the patient” consists of the parents and the child itself [24]. Differences and predictors of dropout in the present study were based on the lowest levels of z-BMI and subscapular skin fold. However, predictors described in other studies were related to parental BMI, distance from home to treatment site, and depressive symptoms [23, 25]. Other barriers to achieve physical activity in children at a hospital setting mentioned in another study were financial issues/limitations, the fact that this type of intervention was not a priority, and lack of physical space [26].

Although many systematic reviews provide information about diet and exercise intervention to reduce BMI [9-11], just a few studies have been conducted under real clinical practice conditions. Weight loss in pediatric outpatients is a difficult outcome to achieve; however, small weight reductions can produce health-related improvements in other cardiovascular risk factors [27]. Implementing joint parent-child interventions may be a better method to achieve risk factor reductions in the clinical context [28]. To improve compliance, some authors suggest providing motivation at the beginning of the intervention, using telephone calls or short message service (SMS), age matched groups, and avoiding intensive or everyday interventions because these goals may be too challenging or unrealistic for this population [28-32]. However, most of the studies conducted so far were non-randomized or selected specific populations (such as highly motivated volunteers), which limits the use of these findings in routine clinical care.

Additionally to the already mentioned limitation of our small sample size, other limitations should be mentioned. It is not possible to blind some types of interventions, including physical activity and exercise interventions, and placebo is very difficult to apply in this situation. Improvements in physical activity daily routines can make a difference in biological and anthropometric markers because they change the daily energy intake. In addition, it is impossible to blind a person about how much exercise or physical activity he/she is doing. Similarly, the research team cannot be blinded about what they are advising. Thus, our study was non-blinded for the children and the professionals implementing the intervention. To improve the strength of our study, the outcome assessor was blind to allocation group and we performed intention-to-treat analyses. The RCT methodology was strictly followed. We also tried to measure and to minimize all possible bias and predictors in order to perform statistical analyzes properly. It is important to publish the results of small clinical trials so they can be included in future meta-analysis.

### Conclusion

A low-cost physical activity counseling intervention presented many barriers for implementation in routine clinical care, limiting its feasibility and evaluation of effectiveness to reduce cardiovascular risk factors. New studies incorporating motivational techniques to improve family participation and other resources, such as short message service (SMS) and electronic media, may bring new insights into this issue. However, it is important to keep in mind that the interventions must be simple and inexpensive if they are to be implemented in contexts such as the one described in the present study.
